# A comprehensive review of factors affecting growth and secondary metabolites in hydroponically grown medicinal plants

**DOI:** 10.1007/s00425-025-04619-y

**Published:** 2025-01-30

**Authors:** Neeharika Narisepalli Venkatasai, Devija N. Shetty, Chigateri M. Vinay, Mahendran Sekar, Annamalai Muthusamy, Padmalatha S. Rai

**Affiliations:** 1 Department of Biotechnology, Manipal School of Life Sciences, Manipal Academy of Higher Education, Manipal, India; 2 School of Pharmacy, Monash University Malaysia, Bandar Sunway, Selangor Darul Ehsan Malaysia; 3 Department of Plant Sciences, Manipal School of Life Sciences, Manipal Academy of Higher Education, Manipal, India

**Keywords:** Nutrients, pH, Electrical conductivity, Light, Temperature, Hormonal elicitors, Nanoparticles, Microorganisms

## Abstract

**Main conclusion:**

Optimizing environmental factors can significantly increase the growth and secondary metabolite synthesis of hydroponically grown medicinal plants. This approach can help increase the quality and quantity of pharmacologically important metabolites to enhance therapeutic needs.

**Abstract:**

Medicinal plants are key therapeutic sources for treating various ailments. The increasing demand for medicinal plants has resulted in the overharvesting of these plants in their natural habitat, which can lead to their extinction in the future. Soil-based cultivation faces challenges, such as a lack of arable land, drastic climatic changes, and attacks by soil-borne pathogens. To overcome these challenges, hydroponic cultivation, known as soilless cultivation, is a sustainable method. The yield and quality of medicinal plants depend on environmental factors, such as nutrients, pH, electrical conductivity, temperature, light, nanoparticles, phytohormones, and microorganisms. This article explores the impact of these environmental factors on the growth and secondary metabolite content of hydroponically grown medicinal plants. Our review reveals how environmental factors qualitatively and quantitatively influence the growth and secondary metabolites of medicinal plants grown in hydroponic systems and how these factors can be integrated into the enhancement of therapeutic compounds.

**Graphical abstract:**

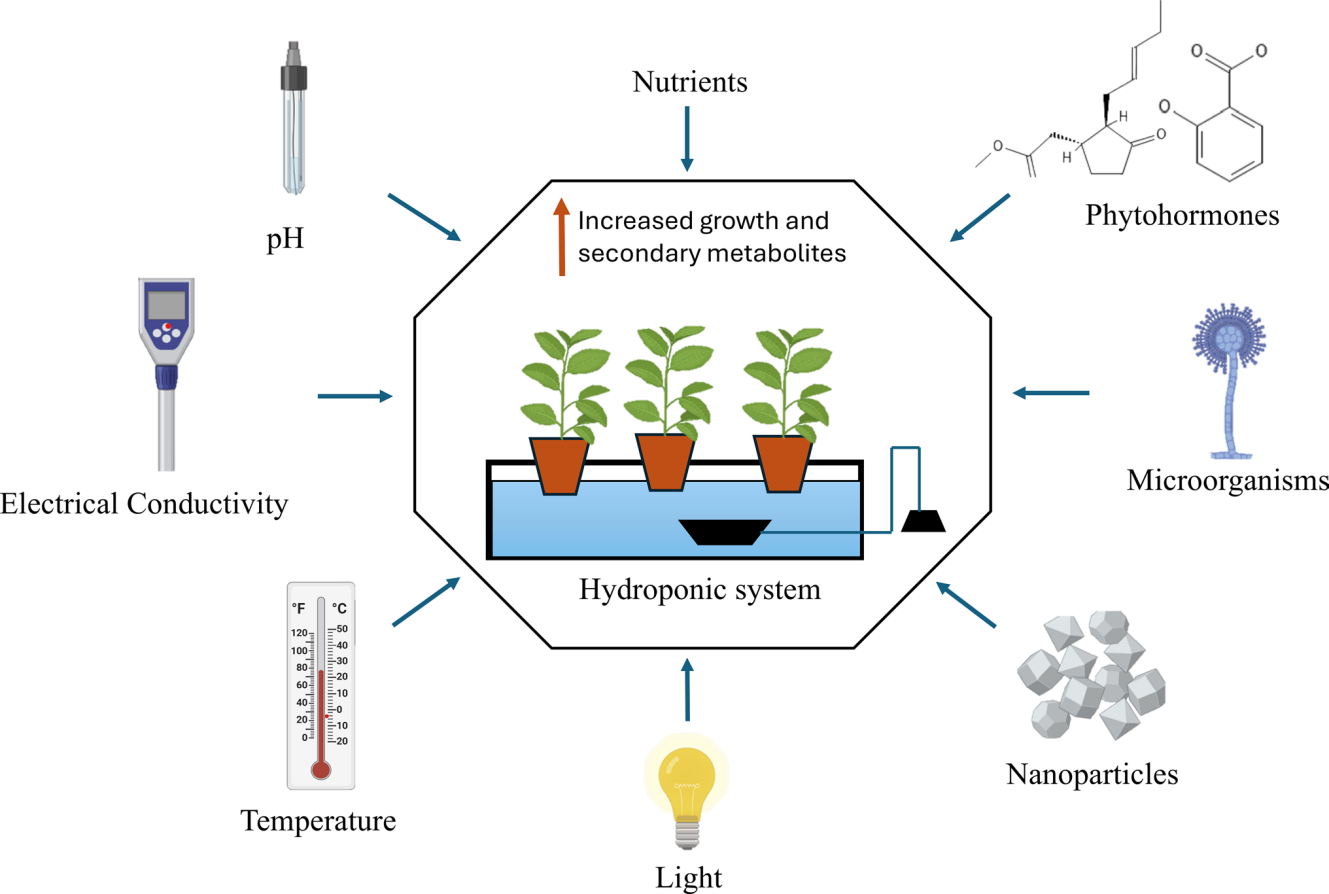

## Introduction

Since ancient times, medicinal plants have played a pivotal role in health care. Even today, 80% of people in developing countries rely on herbal medicines to maintain their health (Asafo-Agyei et al. 2023). In developed countries, 25% of medicines prescribed originate from wild plants (Chen et al. 2016). The medicinal plants contain secondary metabolites, such as flavonoids, tannins, steroids, saponins, and alkaloids, which play important roles in their therapeutic properties. These secondary metabolites can potentially treat diseases and syndromes, such as diabetes, stroke, cancer, Alzheimer’s disease, and Parkinson’s disease (Crozier et al. 2009; Davies and Espley 2013). For instance, *Catharanthus roseus* produces vinblastine and vincristine, which are used in cancer treatment (Rai et al. 2014). Ginsenosides from *Panax ginseng* are used for managing diabetes (Kim 2018). *Ginko biloba* produces ginkgolides used to treat Alzheimer’s and Parkinson’s diseases (Gachowska et al. 2021). Curcumin, a bioactive compound obtained from *Curcumin longa*, has the potential to manage stroke (Almutairi et al. 2022). Recently, secondary metabolites synthesized in plants have been used as raw materials to manufacture drugs. Many plant-based life-saving drugs, such as paclitaxel, camptothecin, combretastatins, vinblastine, and vincristine, are available (Khazir et al. 2014). Some of the pharmaceutically important plants and their secondary metabolites used in therapy are listed in Table 1. The international market for medicinal plants is experiencing significant growth, with a value of 170 billion USD in 2022, which is expected to increase to 600 billion USD in 2033 (Silveira and Boylan 2023). This highlights the increasing demand for medicinal plants in the coming days, resulting in the overexploitation of medicinal plants from natural sources. Therefore, a sustainable method for growing medicinal plants must be employed to address increasing demand (Marcelino et al. 2023).

**Table 1:** Pharmaceutically important plants and their bioactive secondary metabolites

Plant species	Drug	Pharmacological effect	References
*Catharanthus roseus*	Vinblastine, vincristine	Anticancer	Rai et al. (2014)
*Rauwolfia serpentina*	Serpentine, reserpine	Antihypertensive	Kumari et al. (2013)
*Taxus brevifolia*	Taxol	Anticancer	Sze et al. (2008)
*Digitalis lanata*	Digoxin	Cardiotonic	Bhusare et al. (2018)
*Cinchona officinalis*	Quinine	Antimalarial	Yeca et al. (2009)
*Thymus vulgaris*	Thymol	Analgesic	Soković et al. (2008)

Conventional soil-based cultivation has many drawbacks, such as the risk of plants being infected by nematodes, disease-causing microorganisms, and the use of pesticides and chemicals. In addition, conventional soil-based cultivation faces challenges, such as drastic climatic changes, a lack of arable land for agriculture, and water shortages (Barman et al. 2016; Surendran et al. 2017). Hydroponics, known as soilless cultivation, is a sustainable plant-growing method in which plants are grown in nutrient media (liquid culture) or artificial growth media (substrate culture) (Ahmed et al. 2020). Hydroponics offers advantages such as a lack of soil, high yield, and the ability to grow plants under controlled conditions (Khan et al. 2020). Compared with those grown in soil, hydroponics is particularly effective for cultivating high-quality medicinal plants rich in bioactive compounds (Gaja et al. 2023).

**Fig. 1:**
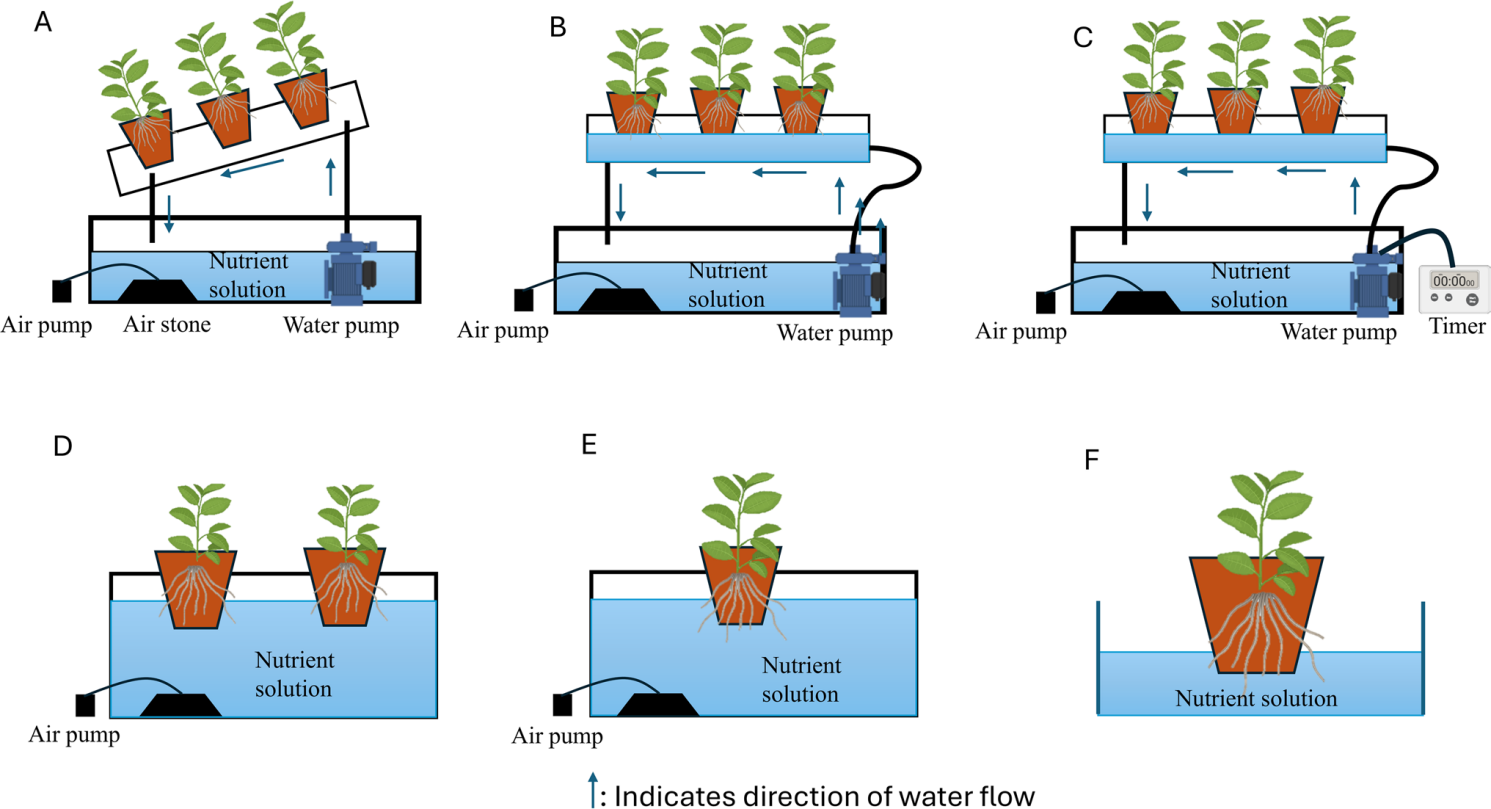
Circulating and non-circulating hydroponic systems. **A** Nutrient film technique.** B** Deep flow technique.** C** Ebb and flow technique. **D** Floating technique **E** Root dipping technique, **F** Capillary action technique

**Fig. 2:**
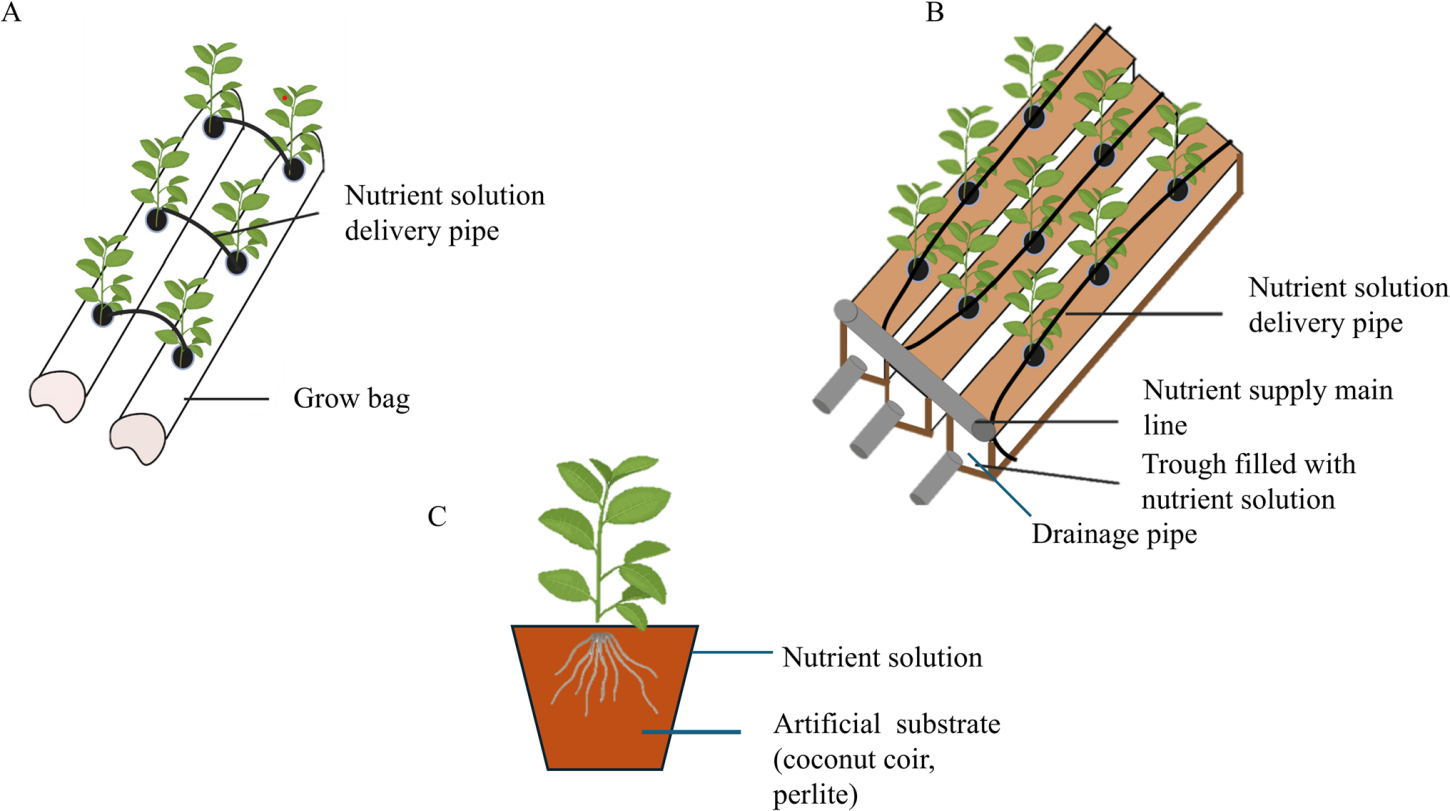
Solid substrate hydroponic systems. **A** Grow bag technique.** B** Trough technique.** C** Pot technique

Different types of hydroponic systems exist, such as circulating, noncirculating, and solid substrate hydroponic systems (Hussain et al. 2014). The basic design of the hydroponic systems is represented in Figs. 1 and 2. The choice of the hydroponic system primarily depends on the type of plant being grown. In circulating systems, nutrient solution circulates from the reservoir to the tank. Examples of circulating hydroponic systems include nutrient film and deep flow techniques. The nutrient film technique uses a sloped tank that allows shallow water to flow over the plant roots, whereas the deep flow technique utilizes a tray filled with nutrient solution, where the roots are submerged. The ebb and flow technique is similar to the deep flow technique, in which nutrient solution is periodically added and drained from a plant growth tray. However, in noncirculating hydroponic systems, the nutrient mixture remains in the tank. Examples of noncirculating methods include root dipping, floating, and capillary action techniques. In the capillary action technique, the nutrient solution is supplied to plants by placing the pot in a container filled with nutrient solutions, and the nutrient solution reaches the plants via capillary action (Atherton and Li 2023). In the floating technique, the roots are entirely submerged, whereas in the root dipping technique, only the bottom portion of the roots is in contact with the nutrient solution (Bringas‐Burgos et al. 2023). In solid media culture, the plants are supported by inert substrates, such as perlite, vermiculite, and coconut coir instead of soil, and the nutrient solution is fertigated at frequent intervals. Grow bag, pot, and trough techniques are examples of solid culture methods. In the grow bag technique, polythene bags are filled with artificial substrate with small holes cut into the bag to place the seedlings inside, and nutrient media is supplied through pipes. The pot technique involves placing plants within individual pots filled with artificial substrates and fertigating them with nutrient media. In the trough technique, troughs are constructed above the ground, lined with waterproof material, and filled with inert media and plants are placed in the troughs, and nutrient media is supplied via a drip system (Atherton and Li 2023; Hussain et al. 2014). These diverse hydroponic systems also increase the production of bioactive compounds with pharmaceutical importance (Gaja et al. 2023). The list of pharmaceutically important drugs that can be produced using hydroponic systems is given in Table 2.

**Table 2:** Pharmaceutically important drugs produced using hydroponic systems

Plant species	Drug	Part used	References
*Catharanthus roseus*	Vindoline and catharanthine	Leaves	Hendrickson et al. (2022)
*Ophiorrhiza pumila*	Camptothecin	Roots	Lee et al. (2020)
*Withania somnifera*	Withaferin	Roots	Von bieberstein et al. (2014)
*Agastache rugosa*	Tilianin	Whole plant	Lam et al. (2020)
*Silybum marianum*	Silymarin	Fruits	Ahmed et al. (2020)
*Glycyrrhiza glabra*	Glycyrrhizin	Roots	Afreen et al. (2005)

In addition to the various hydroponic systems utilized for cultivating medicinal plants, environmental factors are pivotal for influencing plant growth and the production of secondary metabolites. The growth of medicinal plants under controlled environmental conditions increases their biomass, quality, and secondary metabolites (Ćavar Zeljković et al. 2022). Studies have revealed environmental factors, such as mineral nutrients, light, temperature, and microorganisms. significantly influences plant growth and secondary metabolite production (Ramakrishna and Ravishankar 2011; Ahmadi et al. 2021; Fukuyama et al. 2015; Lee et al. 2020; Mubeen et al. 2022; Suksawat and Panichayupakaranant 2024). By carefully manipulating these conditions, growers can cultivate medicinal plants despite climatic changes and ultimately improve the yield and quality of the medicinal plants (Pant et al. 2021). This review focuses on how various environmental factors, such as nutrients, pH, electrical conductivity (EC), temperature, light, nanoparticles, phytohormones, and microorganisms, affect plant growth and secondary metabolite synthesis. This review highlights the role of external factors that may be applied to develop high-quality drug production in a controlled system.

## Methodology

Data for this review were identified by searching databases, such as SCOPUS, PUBMED, and Google Scholar. The terms used were related to environmental factors affecting the growth and secondary metabolites of hydroponically grown medicinal plants. The terms used were hydroponics, hydroponic cultivation, medicinal plants, environmental factors, nutrients, nitrogen, phosphorus, calcium, pH, electrical conductivity, temperature, light, nanoparticles, elicitors, methyl jasmonate, salicylic acid, and microorganisms**.** The search results were reviewed to identify potentially relevant studies. Only original articles, reviews, and book chapters published between 2002 and 2024 were included. Editorials, university theses, and letters were excluded.

## Results

### Factors affecting the growth and secondary metabolites of hydroponically grown medicinal plants

Hydroponics offers precise control of environmental factors, such as nutrients, pH, electrical conductivity, light, temperature, phytohormones, and microorganisms. Controlling these factors allows enhanced plant growth and metabolite production. (Khan et al. 2020; Bae et al. 2017; Meselmani 2022; Kafle et al. 2017; Shawon et al. 2023). For instance, nutrients play a vital role in hydroponic nutrient solutions. The formulation of macronutrients and micronutrients can increase plant growth and metabolite production (Meselmani 2022). pH is a crucial factor for nutrient availability, and variations in pH can result in the precipitation of salts in nutrient solutions, affecting nutrient absorption by plants. Electrical conductivity is an indicator used to evaluate the total ion concentration in nutrient solutions. The light spectrum plays a role in synthesizing secondary metabolites; for example, red light increases phenolic contents in *Crepidiastrum denticulatum* (Bae et al. 2017). Temperature also significantly affects plant growth and secondary metabolite production, with different plant species having varying temperature requirements (Sharma et al. 2022). In addition to these factors, phytohormones and microorganisms increase the production of secondary metabolites, such as flavonoids and phenolic compounds (Mubeen et al. 2021). By carefully regulating these factors, cultivators can produce high-quality medicinal plants. (Meselmani 2022).

### Nutrients

Nutrient management is essential for the successful management of hydroponic systems. Seventeen essential elements are required for plant growth. Carbon, oxygen, hydrogen, nitrogen, potassium, phosphorus, sulphur, magnesium, and calcium are required in relatively large quantities and are referred to as macronutrients. Iron, copper, manganese, zinc, iron, chloride, molybdenum boron, and cobalt are required in small quantities as micronutrients (White and Brown 2010). A hydroponic nutrient solution contains a balanced ratio of macronutrients and micronutrients essential for plant growth (Meselmani 2022).

Nitrogen, potassium, and phosphorus are especially important for plant growth. Nitrogen is crucial for plant growth, as it is a fundamental component of nucleic acids and amino acids (Roosta 2024). In nutrient solutions, the two primary inorganic forms of nitrogen are nitrate (NO₃⁻) and ammonium (NH₄⁺) (Guo et al. 2012). For example, *Echinacea purpurea* is grown hydroponically with different ratios of ammonia and nitrate (90:10 and 70:30). They reported that an increase in the NO_3_^−^/NH_4_^+^ ratio resulted in considerable increases in growth parameters, total phenolic content, and caffeic acid derivatives (Ahmadi et al. 2021). A similar study was conducted by Olfati et al. (2012) to evaluate the growth and yield of *Ocimum basilicum* L. and *Lepidium sativum* L. They reported that an increase in ammonium concentration (1 mEq L^−1^) favoured the growth of *Lepidium sativum* L., whereas an increasing nitrate concentration (8.5 mEq L^−1^) favoured the growth of *Ocimum basilicum* L. (Olfati et al. 2012).

Potassium is vital for carbohydrate, fat, and protein synthesis. It also plays a role in photosynthesis, as well as in the transport of sugar and water nutrients. It significantly affects plant growth, flowering, fruit quality, pest resistance, and secondary metabolite content. Studies have shown that potassium significantly affects essential oil production in *Mentha spicata* L. (Chrysargyris et al. 2017a, b, c). Phosphorus, another macronutrient, is required for physiological functions, such as photosynthesis, enzymatic reactions, and carbohydrate metabolism. It is a structural component of phospholipids, deoxyribonucleic acid, and ribonucleic acid. Plants treated with low phosphorus concentration (5 mg L^−1^) exhibited increased biomass, buds, and capitulum in *Calendula officinalis* L. (Stewart and Lovett-Doust 2003). In addition to nitrogen, potassium, and phosphorus, other nutrients play crucial roles in growth, development, and physiological functions, such as disease resistance, carbohydrate metabolism, protein metabolism, hormone synthesis, enzyme activity, photosynthesis, and maintenance of the plasma membrane structure (Suryawanshi 2021). Therefore, the manipulation of macronutrient and micronutrient compositions plays a key role in plant growth and the synthesis of secondary metabolites. A summary of the effects of nutrients on plant growth and metabolite content is given in Table 3. This information can guide cultivators in designing a strategy to increase the growth and secondary metabolites of hydroponically grown medicinal plants.

**Table 3:** Effect of nutrients on growth and secondary metabolites in hydroponically grown medicinal plants

Plant species	Nutrients	Technique	Effect on growth	Effect on secondary metabolites	References
*Chrysanthemum coronarium* L	Nitrogen (ammonium ion)	Deep flow technique	Optimum flowerhead yield (3.1 mM of ammonium)	Increased sesquiterpene lactones in the flowerheads (3.1 mM ammonium ion concentration)	Yang et al. (2005)
*Lavendula angustifolia* Mill	Potassium	Pot technique	41% increase in root development (325, 350 mg L^−1^ of potassium)	Increased phenols and flavonoids in leaves (At 300, 325 and 350mgL^−1^ potassium concentration)	Chrysargyris et al. (2017a, b, c)
*Calendula officinalis* L	Phosphorus	Deep flow technique, floating technique and pot technique	Increased leaf biomass under high phosphorus treatment (200 mg L^−1^ of phosphorus); Increased inflorescence under low phosphorus treatment (5 mg L^−1^ of phosphorus)	NA	Stewart and Lovett-Doust (2003)
*Chrysanthemum coronarium* L	Calcium		Maximum leaf yield (30.9 mM of calcium) and flower yield (38.0 mM of calcium)	71% increase in sesquiterpene lactone contents in flowers (40 mM of calcium)	Supanjani et al. (2005)
*Mentha spicata* L	Nitrogen	1L container with nutrient solution	Increased biomass at (200 mg L^−1^ nitrogen concentration)	Increased levels of carvone, limonene, 1,8-cineole, germacrene D and β-pinene in the leaves and stems (225 mg L^−1^ of nitrogen)	Chrysargyris et al. (2017a, b, c)
*Mentha spicata* L	Phosphorus	Deep flow technique	Increased dry matter (70 mg L^−1^ of phosphorus)	Increased rosmarinic acid in aerial plant parts (70 mg L^−1^ of phosphorus)	Chrysargyris et al. (2019)
*Lavandula angustifolia* Mill	Nitrogen and phosphorus	Pot technique	Increased root biomass (200 mg L^−1^ of nitrogen); Increased plant biomass (70 mg L^−1^ of phosphorus)	Increased 1.8-cineole (200 mg L^−1^ of nitrogen and 60 mg L^−1^ of phosphorus) borneol, camphor (150 mg L^−1^ of nitrogen) α-terpineol (50 mg L^−1^ of phosphorus) myrtenal (150 mg L^−1^) in leaves	Chrysargyris et al. (2016)
*Origanum dictamnus* L	Phosphorus	Nutrient film technique	NA	Increased essential oil content in leaves (5 mg L^−^ samples ^1^ of phosphorus) Increased essential oil content in bracts (60 mg L^−1^ of phosphorus) *p*-cymene and carvacrol are main compounds identified (5 mg, 30 mg and 60 mg L^−1^ phosphorus)	Economakis et al. (2002)
*Salvia officinalis* L	Nitrogen	12L container filled with nutrient solution	Increased plant height, leaf fresh and dry weight, leaf area, number of branches, root fresh and dry weight (14.6 mM of nitrogen)	Increased carotenoids, proline, total phenolic content, oxygenated monoterpenes in leaves (14.6 mM of nitrogen); increased α-thujone at (10 mM of nitrogen)	Khammar et al. (2021)
*Prunella vulgaris* L	Phosphorus	5L plastic container filled with nutrient solution	Increased spica number (15.60 ± 3.03), spica length (3.74 ± 0.70 cm), shoot height (23.39 ± 2.41 cm), dry weight (5.91 ± 0.33 g plant^−1^), root weight (0.46 ± 0.03 g plant^−1^) under 0.20 mM phosphorus application	Increased oleanolic acid and ursolic acid under 10 mM phosphorus application	Yu et al. (2016)

NA - Not Available

### pH

In addition to nutrients in the hydroponic nutrient solution, pH is critical for optimal plant growth and secondary metabolite production. A nutrient solution with a pH ranging from 5.5 to 6.5 is ideal for plants to absorb nutrients. In this pH range, all nutrients, such as nitrogen, phosphorus, potassium, sulphur, magnesium, calcium, iron, boron manganese, copper, and zinc, are readily available to plants (Lu and Shimamura 2018)**.** An increase in pH above 7.5 decreases the availability of phosphorus, manganese, iron, boron, zinc, and copper. A pH below 5 decreases the availability of magnesium, zinc, calcium, and copper (Ncise et al. 2021). These findings indicate that a change in pH significantly affects the availability of nutrients to plants.

pH significantly affects plant growth and nutrient uptake in medicinal plants. A low pH (pH 4) increases growth and enhances the production of carotenoids, phenols, and chlorophyll in *Taraxacum officinale (L.)* Weber ex F.H. Wigg. (Alexopoulos et al. 2021). A neutral pH of 7, on the other hand, has increased the biomass in *Stevia* (Kafle et al. 2017). The effects of pH on growth and secondary metabolite production are shown in Table 4.

**Table 4:** Effect of pH on growth and secondary metabolites in hydroponically grown medicinal plants

Plant species	pH ranges	Type of Hydroponic system	Effect on growth	Effect on metabolites	References
*Artemisia afra*	4.5, 5.5, 6.5, 7.5, 8.5	Pot technique	Fresh weight was highest at pH 6.5; decrease in dry weight at pH 4.5, 8.5	NA	Koehorst et al. (2010)
*Taraxacum officinale*	4.0, 5.5, 7.0	Containers filled with nutrient solution	Increased leaves (29.9), rosette diameter (45.8 cm) at pH 5.5	Enhanced total phenolics (758.4 mg GAE kg^−1^ fresh weight) carotenoids (105.9 mg kg^−1^ fresh weight) and chlorophyll in leaves at pH 4.0	Alexopoulos et al. (2021)
*Reichardia picroides*	4.0, 5.5, 7.0	Containers filled with nutrient solution	Increased leaves (68.9), rosette diameter (44.0 cm) at pH 5.5	Enhanced total phenolics (1214.8 mg gallic acid equivalents kg^−1^ fresh weight) carotenoids (79.7 mg kg^−1^ fresh weight) at pH 4.0	Alexopoulos et al. (2021)
*Stevia rebaudiana*	4.0, 5.0, 6.0, 7.0, 8.0	Styrofoam boxes (53 cm × 23 cm × 25 cm) filled with nutrient solution	Increased total above-ground biomass (27.9 g/plant) at pH 6	2.97% increase in steviol glycosides in leaves at pH 7.0	Kafle et al. (2017)

NA - Not Available

Controlling the pH of the nutrient solution is critical for plant development and nutrient availability. Furthermore, changes in pH might alter secondary metabolite synthesis in particular plant species, highlighting the need for pH regulation for specific therapeutic plants. Understanding and controlling pH levels in hydroponic systems is critical for optimizing plant growth, nutrient absorption, and metabolite synthesis.

### Electrical conductivity

The electrical conductivity of a nutrient solution is a crucial factor required for plant growth and nutrient uptake. The electrical conductivity is a measure of the total number of ions in the nutrient solution. An optimum electrical conductivity ranging from 1.5 to 2.5 dS m^−1^ is required for plant growth (Samarakoon et al. 2006). Deviations from this range can have adverse effects: high electrical conductivity causes salt stress, whereas low electrical conductivity leads to nutrient deficiency in plants (Flores-Sánchez et al. 2023). Electrical conductivity within the optimum range increased centelloside production in *Centella asitatica* (Shawon et al. 2023). High electrical conductivity (8.5 dS m⁻^1^) reduces flavonoid, phenol, and antioxidant contents, as well as the uptake of minerals, such as nitrogen, potassium, phosphorus, and magnesium while increasing the uptake of calcium and potassium. (Chrysargyris et al. 2021). The combined effect of electrical conductivity and the hormonal elicitor methyl jasmonate enhances the synthesis of acacetin (electrical conductivity of 2.0 dS m^−1^, methyl jasmonate of 10 µM) and tilianin (electrical conductivity of 4.0 dS m^−1^, methyl jasmonate of 20 µM) in *Agastache rugosa* (Kim et al. 2018). The influence of electrical conductivity on medicinal plant growth and secondary metabolite production is given in Table 5. Therefore, the optimization of electrical conductivity is essential for obtaining high yields and secondary metabolite production in medicinal plants.

**Table 5:** Effect of electrical conductivity on growth and secondary metabolites in hydroponically grown medicinal plants

Plant species	Electrical conductivity (dS m^−1^)	Type of hydroponic system	Effect on growth	Effect on metabolites	References
*Centella asiatica*	0.6, 1.2, 1.8, 2.4 dS m^−1^	Floating technique	Increased growth parameter at 1.2 dS m^−1^ (number of leaves, leaf area, runners, shoot fresh and dry weight) decreased growth parameters at 2.4 dS m^−1^	Increased asiaticoside (1.7 mg g^−1^dry weight), asiatic acid (6.3 mg g^−1^dry weight), madecassoside (11 mg g^−1^dry weight), madecassic acid (36.6 mg g^−1^dry weight) in leaves at 1.2 dS m^−1^	Shawon et al. (2023)
*Agastache rugosa*	0.5, 1.0, 2.0, 4.0, 6.0, 8.0 dS m^−1^	Deep flow technique	Increased leaf length, leaf width, leaf area, stem length, root length, shoot fresh weight, root fresh weight, shoot dry weight, root dry weight at 2.0 and 4.0 dS m^−1^	Increased rosmarinic acid content at 0.5, 1.0, 2.0, 4.0; tilianin at 4.0 acacetin at 0.5 dS m^−1^	Lam et al. (2020)
*Acmella oleracea*	0.5, 1.0, 2.0, 2.5, 3.0, 4.0 dS m^−1^	Pot technique	Improved growth (close to 3.5 mS cm^−1^)	Caryophyllene (6.18% ± 3.25%), (*Z*,*Z*)-1,8,11-heptadecatriene(2.29% ± 0.35%), 1-pentadecene (12.20% ± 2.61%), kessane (3.91% ± 1.22%), spilanthol isomer 1 (2.36% ± 1.18%), (E)-β-ionone (0.56% ± 0.32%), and caryophyllene oxide (4.77% ± 1.08%)	Carmo et al. (2024)
*Ocimum basilicum*	2.0, 1.2, 0.9, 0.7, 0.5 dS m^−1^	Ebb and flow	Growth parameters were significantly higher at EC 1.2 dS m^−1^ and 0.9 dS m^−1^ compared to other treatments	Not available	Hosseini et al. (2021)
*Pelargonium graveolens*	2.1 dS m^−1^ (control) 8.5 dS m^−1^ (high-salinity nutrient solution) 8.5 dS m^−1^ (high salinity concentrated nutrient solution)	Pot technique	Decreased height (39.38 ± 1.36 cm), decreased leaf number (36.00 ± 0.44), fresh weight (48.70 ± 3.37 g), dry weight under high salinity (7.42 ± 0.49) concentrated nutrient solution	High salinity resulted decreased total phenolic and flavonoid contents (8.5 dS m^−1^)	Chrysargyris et al. (2021)
*Verbena officinallis*	2.1 dS m^−1^ (control) 8.5 dS m^−1^ (high-salinity nutrient solution) 8.5 dS m^−1^ (high salinity concentrated nutrient solution)	Pot technique	Decreased height (17.40 ± 0.18 cm), decreased leaf number (42.66 ± 4.75), fresh weight (2.86 ± 0.24 g), dry weight under high salinity (0.50 ± 0.05) concentrated nutrient solution	High salinity resulted decreased total phenolic and flavonoid contents (8.5 dS m^−1^)	Chrysargyris et al. (2021)

### Light

Light regulates plant growth and development and influences plant behavior through photomorphogenesis (Paradiso and Proietti 2022). Light has a significant effect on secondary metabolite synthesis in plants. Plants absorb a specific wavelength of light ranging from 400 to 700 nm (Liu and Iersel 2021). In controlled agricultural systems, fluorescent lamps and high-intensity discharge have been replaced by light-emitting diodes as sources of artificial lighting. Full-spectrum light, blue, red, far red, green, and ultraviolet light are used in controlled environment agriculture (Dou et al. 2017). Studies have shown that red light increases the production of secondary metabolites with pharmacological activity (Fukuyama et al. 2015; Karimi et al. 2022). For example, red light increases amaranthine and vindoline contents in *Catharanthus roseus* L. (Fukuyama et al. 2015). A combination of lights has different effects on plants; for example, *Ocimum basilicum* L. was grown under three different illuminating conditions. *Ocimum basilicum* L. plants grown in red, far-red, and blue light presented a fold increase in yield (Rahman et al. 2021). The effects of different light spectra on hydroponically grown medicinal plants are given in Table 6.

**Table 6:** Effect of light on growth and secondary metabolites in hydroponically grown medicinal plants

Plant species	Light source	Type of hydroponic system	Effect on growth	Effect on secondary metabolites	References
*Catharanthus roseus*	Red light (600 nm)	Plastic container (10L) with nutrient solution	Increased leaf weight at 300 µmol m^−2^ s^−1^ PPF	Increased catharanthine and vindoline contents in leaves at 150 µmol m^−2^ s^−1^ PPF	Fukuyama et al. (2015)
*Hypericum perforatum*	Red (660 nm) and blue light (470 nm)	Nutrient film technique	Increased growth parameters, such as increased flowers, roots and foliage; under red 100% LED (150 µmol m^−2^ s^−1^ PPF); 100% blue LED (150 µmol m^−2^ s^−1^ PPF) resulted in growth retardation	Increased hypericin (4.42%/m^2^), hyperforin (50.67%/m^2^), pseudohypericin (8.07%/m^2^) in flowers under red light; increased proline under blue light	Karimi et al. (2022)
*Ocimum basilicum*	White. Red (680 nm) and blue (460 nm) light	Pot technique	Onefold increase in yield under blue, and far red light	NA	Rahman et al. (2021)
*Geranium thunbergii*	Red light (660 nm)	Nutrient film technique	Decreased fresh weight, above ground fresh and dry weight	Geraniin content was highest in leaves under red light (70 mg g^−1^dry weight)	Watanabe et al. (2011)
*Panax ginseng*	Red light (630–700 nm) and yellow light	Deep flow technique	Increased biomass	NA	Kim et al. (2023)
	White light	Deep flow technique	Increased root biomass	NA	Kim et al. (2023)
*Lippia palmeri*	Full spectrum LED light	Floating raft hydroponic system	Increased height (37.4 ± 19.7 cm), leaf length (5.2 ± 1.4 cm) and width (2.6 ± 0.6 cm), number of shoots (15.5 ± 12.6), canopy diameter (53.4 ± 15.9 cm), leaf dry weight (41.8 ± 8.0 g)	47% increased carvacrol content in leaves	Bringas‐Burgos et al. (2023)
*Crepidiastrum denticulatum*	Red (660 nm)/Far-red light (735 nm)	Pot technique	Leaf length, leaf area, shoot fresh, dry weight was 1.8–2.4 times higher under red/far red light ratio of 0.7 and 1.2	1.3–1.8-fold increase in chlorogenic acid, caffeic acid, and chicoric acid levels per shoot under red/far red light ratio of 0.7 and 1.2	Bae et al. (2017)
*Glycyrrhiza* *uralensis*	UV B radiation 289–315 nm	Deep flow technique	NA	Increased glycyrrhizin content in roots (at low-intensity UV-B radiation (15 days at 0.43 W m^−2^) at 300 μmol m^−2^ s^−1^ PPF)	Afreen et al. (2005)

NA - Not Available

In addition to the light spectrum, the intensity of light also affects growth and metabolites. Ncise et al. (2020) investigated the influence of light intensity and watering interval on the growth and antifungal activity of hydroponically grown *Tulbaghia violacea* L. plants. They reported that plants grown under 0% shade and 5-day watering intervals presented high fresh and dry weights. They also reported that plants grown in 40% shade with 21-day watering time intervals presented increased antifungal activity (Ncise et al. 2020). Similarly, a study was performed to investigate the influence of low light intensity on *Glycyrrhiza uralensis*. Low light intensity increases the leaf area and concentration of chlorophyll, liquidity, and glycyrrhizic acid but decreases biomass, leaf thickness, and photosynthesis (Hou et al. 2010).

The light spectrum and intensity play crucial roles in regulating plant growth and secondary metabolite production in hydroponically grown medicinal plants. (Hou et al. 2010; Fukuyama et al. 2015) Controlling the illuminating conditions offers a promising solution for growing high-quality plants throughout the year irrespective of climatic conditions (Darko et al. 2014).

### Temperature

Air temperature is essential for understanding the growth of plants. Physiological processes such as respiration, photosynthesis, and transpiration are influenced by temperature. (Hendrickson et al. 2022). For instance, an increase in the temperature increases the yield and photosynthesis rate in rice, whereas in cotton, increasing the temperature reduces photosynthesis. The influence of air temperature on medicinal plants still needs to be explored (Hendrickson et al. 2022). In addition to air temperature, the root zone is also important for physiological factors, such as photosynthesis, nutrient uptake, water uptake, leaf growth, stem growth, and the accumulation of secondary metabolites (Nguyen et al. 2020). An increase in the root zone temperature increases the respiration of roots. Low nutrient solution temperatures induce oxidative stress and reduce plant water uptake (Lee et al. 2020).

Hendrickson et al. (2022) studied the effects of nutrient solution temperature (23 °C, 27.5 °C, and 31 °C) on basil grown in a nutrient film technique system (Hendrickson et al. 2022). They reported that at 27 °C and 31 °C, nutrient solution temperatures resulted in increased growth. Compared with those of the plants grown at 27.5 °C, the width, average leaf area, and fresh and dry weights of the roots and shoots of the plants grown at 31 °C were greater than those of the plants grown at 23 °C (Hendrickson et al. 2022). A study was conducted to investigate the influence of root zone temperature on the growth and camptothecin accumulation of *Ophiorrhiza pumila.* The leaf area, dry and fresh weights, and camptothecin accumulation were highest at a root zone temperature of 20 °C (Lee et al. 2020). Nguyen et al. (2020) also studied the effects of root zone temperature on *Coriandrum sativum* L. They reported that at extreme root zone temperatures (15 °C or 35 °C) for 6 days, the contents of phenolic compounds, ascorbic acid, carotenoids, and chlorogenic acid increased. The effects of temperature on hydroponically grown medicinal plants are shown in Table 7.

**Table 7:** Effect of temperature on growth and secondary metabolites in hydroponically grown medicinal plants

Plant species	Temperature	Technique	Effect on growth	Effect on metabolites	References
*Ocimum basilicum*	23 ℃	Nutrient film technique	Increased width, average leaf area, fresh/dry weight of roots and shoots at 23 °C	NA	Hendrickson et al. (2022)
	27 ℃	Nutrient film technique	Increased growth at 27 ℃	NA	Hendrickson et al. (2022)
	31 ℃	Nutrient film technique	Increased growth, height at 31 ℃	NA	Hendrickson et al. (2022)
*Ophiorrhiza pumila*	20 °C	Container filled with nutrient solution	Highest leaf area (254 cm^2^) dry weight (4.6 g) and fresh weight (30.1 g) at root zone temperature of 20 °C	Highest camptothecin accumulation in the roots (2.0 mg g^-1^) at root zone temperature of 20 °C	Lee et al. (2020)
*Coriandrum sativum*	15 °C or 35 °C	Deep flow technique	Not available	Phenolic compounds, ascorbic acid, carotenoids and chlorogenic acid	Nguyen et al. (2020)

### Nanoparticles

Interest in the use of nanoparticles as abiotic elicitors is increasing because of their ability to regulate plant growth and secondary metabolite production. Their unique physicochemical properties allow them to interact with plants, and nanoparticles can easily be absorbed into plants (Rivero-Montejo et al. 2021). The main advantage is that a low dosage of nanoparticles is sufficient for elicitation. Studies have revealed that nanoparticles, such as copper, zinc and silver nanoparticles increase yield, nutrient uptake, secondary metabolite synthesis, and stress resistance in hydroponically grown medicinal plants (Jadoon et al. 2024; Mubeen et al. 2022; Francis et al. 2022).

The incorporation of nanoparticles into hydroponic systems can significantly increase secondary metabolite synthesis. For instance, silver nanoparticles increase phenolic, flavonoid, nitric oxide, and superoxide dismutase contents in the leaves of hydroponically grown *Silybum marianum* L. (Mubeen et al. 2022). The effects of the nanoparticles on the hydroponically grown medicinal plants are shown in Table 8. The integration of nanoelicitation in hydroponics offers significant economic advantages for the agricultural sector. This advanced approach increases plant growth and the production of secondary metabolites, promoting sustainable cultivation methods.

**Table 8:** Effect of nanoparticles on growth and secondary metabolites in hydroponically grown medicinal plants

Plant species	Type of nanoparticle	Type of hydroponic systems	Treatment method	Effect on growth	Effect on secondary metabolites	References
*Silybum marianum*	Silver nanoparticles (1 ppm)	Floating technique	Nanoparticles added into nutrient solution	NA	Increased phenolics (231 ± 2.4 µg g^−1^) flavonoids (226 ± 0.4 µg g^−1^), content in leaves (1 ppm concentration)	Mubeen et al. (2022)
*Amaranthus hybridus*	Zinc and copper nanoparticles	Container filled with nutrient solution	NA	Increased total fresh weight (66.1 g) and dry weight (8.6 g) and height (0.24 µM of zinc nanoparticles)	NA	Francis et al. (2022)
*Rosmarinus officinalis*	Silver nanoparticles	Container filled with Hoagland solution	NA	NA	11% increase of carnosic acid in leaves (200 ppm of silver nanoparticles)	Hadi Soltanabad et al. (2020)

NA - Not Available

### Phytohormones

Elicitor is a stress factor that directly or indirectly induces a defense mechanism in plants by synthesizing secondary metabolites required to combat stressful conditions (Goel et al. 2011). They are categorized into biotic elicitors, which are of biological origin, and abiotic elicitors, which are nonbiological in origin and include physical, chemical, and hormonal factors. Although hormones are not abiotic stress factors, they are classified as abiotic elicitors, because they induce defense mechanisms in plants. (Naik and Al-Khayri 2016). Elicitation is an economical way to increase the synthesis of secondary metabolites with pharmaceutical importance in medicinal plants (Thakur et al. 2019). Phytohormone elicitors such as salicylic acid and methyl jasmonate effectively increase secondary metabolites in hydroponically grown medicinal plants (Ahmed et al. 2020; Mubeen et al. 2022). Methyl jasmonate is a phytohormonal elicitor that plays a key role in the signalling pathway that regulates the defense response in plants and enhances the synthesis of secondary metabolites (Singh and Dwivedi 2018). Methyl jasmonate increased the production of saponins and phenolic compounds in *Silybum marianum* L., whereas salicylic acid increased the synthesis of flavonolignans in *Silybum marianum* L. (Ahmed et al. 2020; Mubeen et al. 2022). The concentration of the elicitor used also influences the synthesis of secondary metabolites. Wang et al. studied the effects of different concentrations of methyl jasmonate on hydroponically grown *Allium tuberosum Rottl*. The authors reported that 800 µM methyl jasmonate increased the phenolic content of *Allium tuberosum* Rottl (Wang et al. 2022). Phytohormone elicitors and their effects on metabolite production are listed in Table 9. Therefore, choosing the right elicitor(s) and its concentration is important in optimizing the production of the desired secondary metabolite(s) (Wang et al. 2022).

**Table 9:** Effect of phytohormones on growth and secondary metabolites in hydroponically grown medicinal plants

Plant species	Hormonal elicitor	Type of hydroponic system	Effect on growth	Effect on metabolites	References
*Nigella sativa*	Methyl jasmonate	Plastic boxes (10 × 10 × 3.7 cm)	NA	Twelvefold increase in triterpene saponin kalopanaxsaponin in leaves (100 µM of methyl jasmonate application for 7 days)	Scholz et al. (2009)
*Silybum marianum*	Salicylic acid	Nutrient film technique	Higher fruit productivity	1.7-fold accumulation of flavonolignans in fruits (200 mM salicylic acid)	Ahmed et al. (2020)
*Silybum marianum*	Methyl jasmonate (100 mM)	Floating technique	NA	Highest phenolic (372.0 ± 2.4 µg g^−1^) and flavonoids (304 ± 0.3 µg g^−1^) contents in leaves (100 mM Methyl jasmonate)	Mubeen et al. (2022)
*Allium tuberosum*	Methyl jasmonate in different concentrations (0, 300, 500, 800 µM)	Hydroponic boxes (0.37 m × 0.25 m × 0.2 m) filled with nutrient solution	NA	Phenolic contents such as protocatechuic acid (404%) chlorogenic acid (4.1%) are enhanced in leaves (800 µM methyl jasmonate)	Wang et al. (2022)

NA - Not Available

### Microorganisms

Microorganisms are ubiquitous in the environment and have both beneficial and detrimental effects on plant growth. Among them, plant growth-promoting microorganisms are distinctly associated with plant roots, increasing plant growth and providing protection to plants from abiotic stress and diseases (Dhawi et al. 2023). Studies have revealed that plant growth-promoting microorganisms increase secondary metabolite contents in hydroponically grown medicinal plants (Mubeen et al. 2022; Suksawat and Panichayupakaranant 2024). For instance, Mubeen et al. studied the effect of *Aspergillus niger* on hydroponically grown *Silybum marianum* L. and reported an increase in the production of flavonolignans, such as apigenin 7-D glucose, silybin A, silybin B, isosilybin A, and isosilybin B (Mubeen et al. 2021). Similarly, *Trichoderma harzianum* has been found to increase the production of rhinacanthin in the roots of hydroponically grown *Rhinacanthus nasutus* (Suksawat and Panichayupakaranant 2024). However, few studies exist on the role of microorganisms in growth and secondary metabolite production. The effects of microorganisms on hydroponically grown plants are given in Table 10.

**Table 10:** Effect of microorganisms on growth and secondary metabolites in hydroponically grown medicinal plants

Plant species	Microorganism	Type of hydroponic system	Effect on growth	Effect on metabolites	References
*Rhinacanthus nasutus*	*Trichoderma harzianum*	NA	NA	2.2 fold increase in rhinacanthin content	Suksawat and Panichayupakaranant (2024)
*Silybum marianum*	*Aspergillus niger*	Floating technique	NA	High phenolic (282.2 ± 2.5 and µg g^−1^) flavonoids (245 ± 0.4 µg g^−1^) content in the leaves	Mubeen et al. (2022)
*Silybum marianum*	*Aspergillus niger*	Container filled with nutrient solution	NA	High flavonolignans content, such as apigenin 7-D glucose, silynin A, silybin B, isosilybin A, isosilybin B	Mubeen et al. (2021)

NA - Not Available

### Applications of hydroponics systems

Hydroponics has a wide range of applications due to its adaptability and efficiency. Hydroponics is useful in urban settings, where limited space is available, and can be installed in balconies, terraces, and courtyards. In controlled agricultural facilities, hydroponics allows for the precise alteration of environmental conditions, such as light, temperature, humidity, and nutrient supply, leading to optimum plant growth (Meselmani 2022). Hydroponic cultivation is highly valuable for long-term space missions, because it provides fresh food, purifies the air, and balances oxygen and carbon dioxide at space stations. Unlike animal farming, which is not feasible, hydroponics allows the cultivation of the fresh foods required for astronauts. It supports a bioregenerative life support system by absorbing CO2 and releasing O2, which is vital for sustaining long-term habitation at space stations and other planets. Hydroponics offers a versatile and sustainable solution for terrestrial and space food production. (Khan et al. 2020).

Globally, companies are using automated hydroponic systems to grow plants efficiently. In India, startups are employing hydroponics systems to grow vegetables, providing a sustainable alternative to sustainable agriculture (Khan et al. 2020).

### Recent advancements in hydroponics

There is an increasing demand for medicinal plants worldwide. Sustainable methods such as hydroponics must be employed to prevent the exploitation of these medicinal plants in their natural habitat (Marcelino et al. 2023). Recent advancements in hydroponics have utilized computational methods in which sensor technology is integrated with computational methods. The computational method uses the Internet of Things, machine learning algorithms, and deep neural network technologies. These technologies facilitate the automation of hydroponics systems by monitoring pH, EC, and water levels, and their information can be stored in the cloud (Srivastava and Mathur 2023). Furthermore, IoT technology can also be employed in hydroponics for disease and pest detection to improve overall growth by deploying countermeasures (de Abreu and van Deventer 2022). Despite its numerous benefits, hydroponics has several limitations. High capital investment is required to establish hydroponic systems. In addition, technical expertise is necessary to effectively maintain these systems. However, alternative hydroponic systems, such as aquaponics and aeroponics, offer some solutions. **(**Velazquez-Gonzalez et al. 2022).

## Conclusion and future prospectives

The current study highlights the impact of environmental factors such as nutrients, pH, EC, light, temperature, nanoparticles, phytohormones, and microorganisms that substantially affect the growth and secondary metabolite composition of hydroponically grown medicinal plants. Optimizing these parameters can significantly affect the growth, quantity and quality of the secondary metabolites synthesized. This knowledge can be applied to increase the commercial production of medicinal plants with greater quality and quantity of pharmacologically important metabolites. Moreover, it is also a sustainable solution to prevent the future extinction of plants in their natural habitat. Hydroponics under controlled environmental conditions is sustainable for meeting the rising demand for medicinal plants in the global market.

## Data Availability

No data sets were generated or analysed during the study.

## References

[CR1] Afreen F, Zobayed SMA, Kozai T (2005) Spectral quality and UV-B stress stimulate glycyrrhizin concentration of *Glycyrrhiza uralensis* in hydroponic and pot system. Plant Physiol Biochem 43:1074–1081. 10.1016/j.plaphy.2005.11.005 16386431 10.1016/j.plaphy.2005.11.005

[CR2] Ahmadi F, Samadi A, Sepehr E, Rahimi A, Shabala S (2021) Optimizing hydroponic culture media and NO^3^^−^/NH^4+^ ratio for improving essential oil compositions of purple coneflower (*Echinacea purpurea* L.). Sci Rep 11:8009. 10.1038/s41598-021-87391-9 33850194 10.1038/s41598-021-87391-9 PMC8044233

[CR3] Ahmed HS, Moawad AS, AbouZid SF, Owis AI (2020) Salicylic acid increases flavonolignans accumulation in the fruits of hydroponically cultured *Silybum marianum*. Saudi Pharm J 28:593–598. 10.1016/j.jsps.2020.03.011 32435140 10.1016/j.jsps.2020.03.011 PMC7229317

[CR4] Alexopoulos AA, Marandos E, Assimakopoulou A, Vidalis N, Petropoulos SA, Karapanos IC (2021) Effect of nutrient solution pH on the growth, yield, and quality of *Taraxacum officinale* and *Reichardia picroides* in a floating hydroponic system. Agron 11:1118. 10.3390/agronomy11061118

[CR5] Almutairi FM, Ullah A, Althobaiti YS et al (2022) A review on therapeutic potential of natural phytocompounds for stroke. Biomedicines 10:2566. 10.3390/biomedicines10102566 36289828 10.3390/biomedicines10102566 PMC9599280

[CR6] Asafo-Agyei T, Appau Y, Barimah KB, Asase A (2023) Medicinal plants used for management of diabetes and hypertension in Ghana. Heliyon 9:e22977. 10.1016/j.heliyon.2023.e22977 38076168 10.1016/j.heliyon.2023.e22977 PMC10703729

[CR7] Atherton HR, Li P (2023) Hydroponic cultivation of medicinal plants—plant organs and hydroponic systems: techniques and trends. Horticulturae 9:349. 10.3390/horticulturae9030349

[CR8] Bae JH, Park SY, Oh MM (2017) Supplemental irradiation with far-red light-emitting diodes improves growth and phenolic contents in *Crepidiastrum denticulatum* in a plant factory with artificial lighting. Hortic Environ Biotechnol 58:357–366. 10.1007/s13580-017-0331-x

[CR9] Barman NC, Hasan MM, Islam MR, Banu NA (2016) A review on present status and future prospective of hydroponics technique. Plant Env Dev 5:1–7

[CR10] Bhusare BP, John CK, Bhatt VP, Nikam TD (2018) In vitro propagation of *Digitalis lanata* Ehrh. through direct shoot regeneration—a source of cardiotonic glycosides. Ind Crops Prod 121:313–319. 10.1016/j.indcrop.2018.05.019

[CR11] Bringas-Burgos BF, Martínez-Robinson KG, Toledano-Magaña Y, García-Ramos JC, Ovando-Martínez M, López-Elías J (2023) Antiproliferative effect of essential oil obtained from oregano (*Lippia palmeri* S. Watson) leaves grown in hydroponics and LED light. Chem Biodivers 20:e202201076. 10.1002/cbdv.202201076 36815541 10.1002/cbdv.202201076

[CR12] Carmo APM, Freitas MSM, Machado LC et al (2024) Electrical conductivity of nutrient solutions affects the growth, nutrient levels, and content and composition of essential oils of *Acmella oleracea* (L.) R. K. Jansen from southeastern Brazil. J Agric Res 15:100968. 10.1016/j.jafr.2024.100968

[CR13] Ćavar Zeljković S, Aucique-Perez CE, Štefelová N, De Diego N (2022) Optimizing growing conditions for hydroponic farming of selected medicinal and aromatic plants. Food Chem 375:131845. 10.1016/j.foodchem.2021.131845 34923398 10.1016/j.foodchem.2021.131845

[CR14] Chen SL, Yu H, Luo HM, Wu Q, Li CF, Steinmetz A (2016) Conservation and sustainable use of medicinal plants: problems, progress, and prospects. Chin Med 11:1–10. 10.1186/S13020-016-0108-7 27478496 10.1186/s13020-016-0108-7 PMC4967523

[CR15] Chrysargyris A, Panayiotou C, Tzortzakis N (2016) Nitrogen and phosphorus levels affected plant growth, essential oil composition and antioxidant status of lavender plant (*Lavandula angustifolia* Mill.). Ind Crops Prod 83:577–586. 10.1016/j.indcrop.2015.12.067

[CR16] Chrysargyris A, Drouza C, Tzortzakis N (2017a) Optimization of potassium fertilization/nutrition for growth, physiological development, essential oil composition and antioxidant activity of *Lavandula angustifolia* Mill. J Soil Sci Plant Nutr 17:291–306. 10.4067/S0718-95162017005000023

[CR17] Chrysargyris A, Nikolaidou E, Stamatakis A, Tzortzakis N (2017b) Vegetative, physiological, nutritional and antioxidant behavior of spearmint (*Mentha spicata* L.) in response to different nitrogen supply in hydroponics. J Appl Res Med Aromat Plants 6:52–61. 10.1016/j.jarmap.2017.01.006

[CR18] Chrysargyris A, Xylia P, Botsaris G, Tzortzakis N (2017c) Antioxidant and antibacterial activities, mineral and essential oil composition of spearmint (*Mentha spicata* L.) affected by the potassium levels. Ind Crops Prod 103:202–212. 10.1016/j.indcrop.2017.04.010

[CR19] Chrysargyris A, Petropoulos SA, Fernandes A, Barros L, Tzortzakis N, Ferreira IC (2019) Effect of phosphorus application rate on *Mentha spicata* L. grown in deep flow technique (DFT). Food Chem 276:84–92. 10.1016/j.foodchem.2018.10.020 30409666 10.1016/j.foodchem.2018.10.020

[CR20] Chrysargyris A, Petropoulos SA, Prvulovic D, Tzortzakis N (2021) Performance of hydroponically cultivated geranium and common verbena under salinity and high electrical conductivity levels. Agron 11:1237. 10.3390/agronomy11061237

[CR21] Crozier A, Jaganath IB, Clifford MN (2009) Dietary phenolics: chemistry, bioavailability and effects on health. Nat Prod Rep 26:1001–1043. 10.1039/b802662a 19636448 10.1039/b802662a

[CR22] Darko E, Heydarizadeh P, Schoefs B, Sabzalian MR (2014) Photosynthesis under artificial light: the shift in primary and secondary metabolism. Philos Trans R Soc B Biol Sci 369:20130243. 10.1098/rstb.2013.0243 10.1098/rstb.2013.0243 PMC3949401 24591723

[CR23] Davies KM, Espley RV (2013) Opportunities and challenges for metabolic engineering of secondary metabolite pathways for improved human health characters in fruit and vegetable crops. N Z J Crop Hortic Sci 41:154–177. 10.1080/01140671.2013.793730

[CR24] De Abreu CL, van Deventer JP (2022) The Application of artificial intelligence (AI) and internet of things (IoT) in agriculture: a systematic literature review. In: Jembere E, Gerber AJ, Viriri S, Pillay A (eds) Artificial intelligence research. Springer, Cham, pp 32–46

[CR25] Dhawi F (2023) The role of plant growth-promoting microorganisms (PGPMs) and their feasibility in hydroponics and vertical farming. Metabolites 13:247. 10.3390/metabo13020247 36837866 10.3390/metabo13020247 PMC9964210

[CR26] Dou H, Niu G, Gu M, Masabni JG (2017) Effects of light quality on growth and phytonutrient accumulation of herbs under controlled environments. Horticulturae 3:36. 10.3390/horticulturae3020036

[CR27] Economakis C, Skaltsa H, Demetzos C, Soković M, Thanos CA (2002) Effect of phosphorus concentration of the nutrient solution on the volatile constituents of leaves and bracts of *Origanum dictamnus*. J Agric Food Chem 50:6276–6280. 10.1021/jf0203444 12381103 10.1021/jf0203444

[CR28] Flores-Sánchez ID, Sandoval-Villa M, Soto-Hernández RM (2023) Effects of electrical conductivity and pruning on secondary metabolite contents in fruits of *Jaltomata procumbens* (Cav.) JL Gentry. Nat Prod Commun 18:1–11. 10.1177/1934578X221150547

[CR29] Francis DV, Sood N, Gokhale T (2022) Biogenic CuO and ZnO nanoparticles as nanofertilizers for sustainable growth of *Amaranthus hybridus*. Plants 11:2776. 10.3390/plants11202776 36297798 10.3390/plants11202776 PMC9610597

[CR30] Fukuyama T, Ohashi-Kaneko K, Watanabe H (2015) Estimation of optimal red-light intensity for production of the pharmaceutical drug components, vindoline and catharanthine, contained in *Catharanthus roseus* (L.) G. Don Environ Control Biol 53:217–220. 10.2525/ecb.53.217

[CR31] Gachowska M, Szlasa W, Saczko J, Kulbacka J (2021) Neuroregulatory role of ginkgolides. Mol Biol Rep 48:5689–5697. 10.1007/s11033-021-06535-2 34245409 10.1007/s11033-021-06535-2 PMC8338821

[CR32] Gaja J, Bala S, Hugara S (2023) Cultivation of medicinal plants using hydroponic system. Int J Res Rev 10:17–21. 10.52403/ijrr.20231003

[CR33] Goel MK, Mehrotra S, Kukreja AK (2011) Elicitor-induced cellular and molecular events are responsible for productivity enhancement in hairy root cultures: an insight study. Appl Biochem Biotechnol 165:1342–1355. 10.1007/S12010-011-9351-7 21909631 10.1007/s12010-011-9351-7

[CR34] Guo XR, Zu YG, Tang ZH (2012) Physiological responses of *Catharanthus roseus* to different nitrogen forms. Acta Physiol Plant 34:589–598. 10.1007/S11738-011-0859-9

[CR35] Hadi Soltanabad M, Bagherieh-Najjar MB, Mianabadi M (2020) Carnosic acid content increased by silver nanoparticle treatment in rosemary (*Rosmarinus officinalis* L.). Appl Biochem Biotechnol 191:482–495. 10.1007/s12010-019-03193-w 31797151 10.1007/s12010-019-03193-w

[CR36] Hendrickson T, Dunn BL, Goad C, Hu B, Singh H (2022) Effects of elevated water temperature on growth of basil using nutrient film technique. HortScience 57:925–932. 10.21273/hortsci16690-22

[CR37] Hosseini H, Mozafari V, Roosta HR, Shirani H, van de Vlasakker PCH, Farhangi M (2021) Nutrient use in vertical farming: Optimal electrical conductivity of nutrient solution for growth of lettuce and basil in hydroponic cultivation. Horticulturae 7:283. 10.3390/horticulturae7090283

[CR38] Hou JL, Li WD, Zheng QY, Wang WQ, Xiao B, Xing D (2010) Effect of low light intensity on growth and accumulation of secondary metabolites in roots of *Glycyrrhiza uralensis* Fisch. Biochem Syst Ecol 38:160–168. 10.1016/j.bse.2009.12.026

[CR39] Hussain A, Iqbal K, Aziem S, Mahato P, Negi AK (2014) A review on the science of growing crops without soil (soilless culture)-a novel alternative for growing crops. Int J Agri Crop Sci 7:833–842

[CR40] Jadoon L, Gul A, Fatima H, Babar MM (2024) Nano-elicitation and hydroponics: a synergism to enhance plant productivity and secondary metabolism. Planta 259:80. 10.1007/s00425-024-04353-x 38436711 10.1007/s00425-024-04353-x

[CR41] Kafle GG, Midmore DJ, Gautam R (2017) Effect of nutrient omission and pH on the biomass and concentration and content of steviol glycosides in stevia (*Stevia rebaudiana* (Bertoni) Bertoni) under hydroponic conditions. J Appl Res Med Aromat Plants 7:136–142. 10.1016/j.jarmap.2017.08.001

[CR42] Karimi M, Ahmadi N, Ebrahimi M (2022) Photoreceptor regulation of *Hypericum perforatum* L. (cv. Topas) flowering under different light spectrums in the controlled environment system. Environ Exp Bot 196:104797. 10.1016/j.envexpbot.2022.104797

[CR43] Khammar AA, Moghaddam M, Asgharzade A, Sourestani MM (2021) Nutritive composition, growth, biochemical traits, essential oil content and compositions of *Salvia officinalis* L. grown in different nitrogen levels in soilless culture. J Soil Sci Plant Nutr 21:3320–3332. 10.1007/S42729-021-00608-8

[CR44] Khan S, Purohit A, Vadsaria N (2020) Hydroponics: current and future state of the art in farming. J Plant Nutr 44(10):1515–1538. 10.1080/01904167.2020.1860217

[CR45] Khazir J, Mir BA, Pilcher L, Riley DL (2014) Role of plants in anticancer drug discovery. Phytochem Lett 7:173–181. 10.1016/j.phytol.2013.11.010

[CR46] Kim J-H (2018) Pharmacological and medical applications of *Panax gingseng* and ginsenosides: a review for use in cardiovascular diseases. J Ginseng Res 42:264–269. 10.1016/j.jgr.2017.10.004 29983607 10.1016/j.jgr.2017.10.004 PMC6026386

[CR47] Kim SJ et al (2018) High electrical conductivity of nutrient solution and application of methyl jasmonate promote phenylpropanoid production in hydroponically grown *Agastache rugosa*. Hortic Sci Technol 36(6):841–852

[CR48] Kim SH, Park JH, Kim EJ, Lee JM, Park JW, Kim YS et al (2023) White LED lighting increases the root productivity of *Panax ginseng* CA Meyer in a hydroponic cultivation system of a plant factory. Biology 12:1052. 10.3390/biology12081052 37626938 10.3390/biology12081052 PMC10452227

[CR49] Koehorst RR, Laubscher CP, Ndakidemi PA (2010) Growth response of *Artemisia afra* Jacq. to different pH levels in a closed hydroponics system. J Med Plant Res 4:1617–1623

[CR50] Kumari R, Rathi B, Rani A, Bhatnagar S (2013) *Rauvolfia serpentina* L. Benth. ex Kurz.: phytochemical, pharmacological and therapeutic aspects. Int J Pharm Sci Rev Res 23:348–355

[CR51] Lam VP, Kim SJ, Park JS (2020) Optimizing the electrical conductivity of a nutrient solution for plant growth and bioactive compounds of *Agastache rugosa* in a plant factory. Agron 10:76. 10.3390/agronomy10010076

[CR52] Lee JY, Hiyama M, Hikosaka S, Goto E (2020) Effects of concentration and temperature of nutrient solution on growth and camptothecin accumulation of *Ophiorrhiza pumila*. Plants 9:793. 10.3390/plants9060793 32630386 10.3390/plants9060793 PMC7355462

[CR53] Liu J, van Iersel MW (2021) Photosynthetic physiology of blue, green, and red light: light intensity effects and underlying mechanisms. Front Plant Sci 12:619987. 10.3389/fpls.2021.619987 33747002 10.3389/fpls.2021.619987 PMC7977723

[CR54] Lu N, Shimamura S (2018) Protocols, issues and potential improvements of current cultivation systems: the next generation indoor vertical farms. In: Kozai T (ed) Smart plant factory. Springer, Singapore, pp 31–49

[CR55] Marcelino S, Hamdane S, Gaspar PD, Paço A (2023) Sustainable agricultural practices for the production of medicinal and aromatic plants: evidence and recommendations. Sustainability 15:14095. 10.3390/su151914095

[CR56] Meselmani MAA (2022) Nutrient solution for hydroponics. In: Turan M, Argin S, Yildirim E, Güneş A (eds) Recent research and advances in soilless culture. IntechOpen, London

[CR57] Mubeen B, Ali Q, Hasnain A, Malik A (2021) Enrichment of therapeutically significant flavonolignans of *Silybum marianum* in vegetative parts by applying fungal elicitors, methyl jasmonate and silver nanoparticles as elicitor in hydroponic culture. J Pharm Res Int 33:126–138. 10.9734/jpri/2021/v33i40B32272

[CR58] Mubeen B, Hasnain A, Mehboob R, Rasool R et al (2022) Hydroponics and elicitation, a combined approach to enhance the production of designer secondary medicinal metabolites in *Silybum marianum*. Front Plant Sci 13:897795. 10.3389/fpls.2022.897795 36035667 10.3389/fpls.2022.897795 PMC9399754

[CR59] Naik PM, Al-Khayri JM (2016) Abiotic and biotic elicitors-role in secondary metabolites production through in vitro culture of medicinal plants. In: Shanker AK, Shanker C (eds) Abiotic and biotic stress in plants—recent advances and future perspectives, vol 17. IntechOpen, London, pp 247–277

[CR60] Ncise W, Daniels CW, Nchu F (2020) Effects of light intensities and varying watering intervals on growth, tissue nutrient content and antifungal activity of hydroponic cultivated *Tulbaghia violacea* L. under greenhouse conditions. Heliyon 6:e03906. 10.1016/j.heliyon.2020.e03906 32455173 10.1016/j.heliyon.2020.e03906 PMC7235940

[CR61] Ncise W, Daniels CW, Etsassala NG, Nchu F (2021) Interactive effects of light intensity and pH on growth parameters of a bulbous species (*Tulbaghia violacea* L.) in hydroponic cultivation and its antifungal activities. Med Plants Int J Phytomed Related Indus 13:442–451. 10.5958/0975-6892.2021.00050.2

[CR62] Nguyen DT, Lu N, Kagawa N, Kitayama M, Takagaki M (2020) Short-term root-zone temperature treatment enhanced the accumulation of secondary metabolites of hydroponic coriander (*Coriandrum sativum* L). grown in a plant factory. Agronomy 10:413

[CR63] Olfati JA, Khasmakhi-Sabet SA, Shabani H (2012) Nutrient solutions on yield and quality of basil and cress. Int J Veg Sci 18:298–304. 10.1080/19315260.2011.642475

[CR64] Pant P, Pandey S, Dall’Acqua S (2021) The influence of environmental conditions on secondary metabolites in medicinal plants: a literature review. Chem Biodiversity 18:e2100345. 10.1002/cbdv.202100345 10.1002/cbdv.202100345 34533273

[CR65] Paradiso R, Proietti S (2022) Light-quality manipulation to control plant growth and photomorphogenesis in greenhouse horticulture: the state of the art and the opportunities of modern LED systems. J Plant Growth Regul 41:742–780. 10.1007/s00344-021-10337-y

[CR66] Rahman MM, Vasiliev M, Alameh K (2021) LED Illumination spectrum manipulation for increasing the yield of sweet basil (*Ocimum basilicum* L.). Plants 10:344. 10.3390/plants10020344 33670392 10.3390/plants10020344 PMC7917910

[CR67] Rai V, Tandon PK, Khatoon S (2014) Effect of chromium on antioxidant potential of *Catharanthus roseus* varieties and production of their anticancer alkaloids: vincristine and vinblastine. Biomed Res Int 2014:934182. 10.1155/2014/934182 24734252 10.1155/2014/934182 PMC3966348

[CR68] Ramakrishna A, Ravishankar GA (2011) Influence of abiotic stress signals on secondary metabolites in plants. Plant Signal Behav 6:1720–1731. 10.4161/psb.6.11.17613 22041989 10.4161/psb.6.11.17613 PMC3329344

[CR69] Rivero-Montejo SDJ, Vargas-Hernandez M, Torres-Pacheco I (2021) Nanoparticles as novel elicitors to improve bioactive compounds in plants. Agriculture 11:134. 10.3390/agriculture11020134

[CR70] Roosta HR (2024) The responses of pepper plants to nitrogen form and dissolved oxygen concentration of nutrient solution in hydroponics. BMC Plant Biol 24:1–12. 10.1186/S12870-024-04943-7 38614965 10.1186/s12870-024-04943-7 PMC11015634

[CR71] Samarakoon UC, Weerasinghe PA, Weerakkody WAP (2006) Effect of electrical conductivity (EC) of the nutrient solution on nutrient uptake, growth and yield of leaf lettuce (*Lactuca sativa* L.) in stationary culture. Trop Agric Res 18:13–21

[CR72] Scholz M, Lipinski M, Leupold M, Luftmann H et al (2009) Methyl jasmonate induced accumulation of kalopanaxsaponin I in *Nigella sativa*. Phytochem 70:517–522. 10.1016/j.phytochem.2009.01.018 10.1016/j.phytochem.2009.01.018 19282005

[CR73] Sharma A, Manpoong C, Devadas VS, Kartha BD, Pandey H, Wangsu M (2022) Crop hydroponics, phyto-hydroponics, crop production, and factors affecting soilless culture. ACS Agric Sci Technol 2(6):1134–1150. 10.1021/acsagscitech.2c00243

[CR74] Shawon MRA, Azad MOK, Ryu BR, Na JK, Choi KY (2023) The electrical conductivity of nutrient solution influenced the growth, centellosides content and gene expression of *Centella asiatica* in a hydroponic system. Agriculture 13:2236. 10.3390/agriculture13122236

[CR75] Silveira D, Boylan F (2023) Medicinal plants: advances in phytochemistry and ethnobotany. Plants 12:1682. 10.3390/plants12081682 37111904 10.3390/plants12081682 PMC10142643

[CR76] Singh A, Dwivedi P (2018) Methyl-jasmonate and salicylic acid as potent elicitors for secondary metabolite production in medicinal plants: a review. J Pharmacogn Phytochem 7:750–757

[CR77] Soković M, Glamočlija J, Ćirić A, Kataranovski D, Marin PD, Vukojević J, Brkić D (2008) Antifungal activity of the essential oil of *Thymus vulgaris* L. and thymol on experimentally induced dermatomycoses. Drug Dev Ind Pharm 34:1388–1393. 10.1080/03639040802130053 18651285 10.1080/03639040802130053

[CR78] Srivastava U, Mathur A (2023) Recent advancements in prevalent practices for plant cultivation by hydroponics. Def Life Sci J 8:255–268. 10.14429/dlsj.8.18781

[CR79] Stewart CL, Lovett-Doust L (2003) Effect of phosphorus treatment on growth and yield in the medicinal herb *Calendula officinalis* L. (Standard Pacific) under hydroponic cultivation. Can J Plant Sci 83:611–617

[CR80] Suksawat T, Panichayupakaranant P (2024) Enhanced rhinacanthin production in *Rhinacanthus nasutus* roots using a hydroponics and elicitation system. J Young Pharm 16:216–222. 10.5530/jyp.2024.16.28

[CR81] Supanjani TARM, Yang MS, Han HS, Lee KD (2005) Calcium effects on yield, mineral uptake and terpene components of hydroponic *Chrysanthemum coronarium* L. Int J Bot 1:146–151. 10.3923/ijb.2005.196.200

[CR82] Surendran U, Chandran C, Joseph EJ (2017) Hydroponic cultivation of *Mentha spicata* and comparison of biochemical and antioxidant activities with soil-grown plants. Acta Physiol Plant 39:1–14. 10.1007/s11738-016-2320-6

[CR83] Suryawanshi YC (2021) Hydroponic cultivation approaches to enhance the contents of the secondary metabolites in plants. In: Shahnawaz M (ed) Biotechnological approaches to enhance plant secondary metabolites. CRC Press, Boca Raton, pp 71–88

[CR84] Sze DM, Miller K, Neilan B (2008) Development of taxol and other endophyte produced anti-cancer agents. Recent Pat Anticancer Drug Discov 3:14–19. 10.2174/157489208783478685 18289120 10.2174/157489208783478685

[CR85] Thakur M, Bhattacharya S, Khosla PK, Puri S (2019) Improving production of plant secondary metabolites through biotic and abiotic elicitation. J Appl Res Aromat Plants 12:1–12. 10.1016/j.jarmap.2018.11.004

[CR86] Velazquez-Gonzalez RS, Garcia-Garcia AL, Ventura-Zapata E, Barceinas-Sanchez JDO, Sosa-Savedra JC (2022) A review on hydroponics and the technologies associated for medium-and small-scale operations. Agriculture 12:646. 10.3390/agriculture12050646

[CR87] Von Bieberstein P, Xu Y, Gunatilaka AAL, Gruener R (2014) Biomass production and withaferin a synthesis by *Withania somnifera* grown in aeroponics and hydroponics. HortScience 49:1506–1509. 10.21273/hortsci.49.12.1506

[CR88] Wang C, Zhang J, Lv J, Li J, Gao Y, Patience BE et al (2022) Effect of methyl jasmonate treatment on primary and secondary metabolites and antioxidant capacity of the substrate and hydroponically grown Chinese chives. Front Nutr 9:859035. 10.3389/fnut.2022.859035 35449536 10.3389/fnut.2022.859035 PMC9016137

[CR89] Watanabe H, Namiki K, Nemoto S, Tajima M, Ono E, Amaki W (2011) Effects of light qualities on geraniin production of *Geranium thunbergii*. Acta Hortic 907:111–114. 10.17660/actahortic.2011.907.13

[CR90] White PJ, Brown PH (2010) Plant nutrition for sustainable development and global health. Ann of Bot 105:1073–1080. 10.1093/aob/mcq085 20430785 10.1093/aob/mcq085 PMC2887071

[CR91] Yang MS, Tawaha AM, Lee YD (2005) Effects of ammonium concentration on the yield, mineral content and active terpene components of *Chrysanthemum coronarium* L. in a hydroponic system. Res J Agric Biol Sci 1:170–217

[CR92] Yeka A, Achan J, D’Alessandro U, Talisuna AO (2009) Quinine monotherapy for treating uncomplicated malaria in the era of artemisinin-based combination therapy: an appropriate public health policy? Lancet Infect Dis 9(7):448–452. 10.1016/S1473-3099(09)70109-4 19555904 10.1016/S1473-3099(09)70109-4

[CR93] Yu M, Chen Y, Zhu Z, Liu L, Zhang L, Guo Q (2016) Effect of phosphorus supply on plant productivity, photosynthetic efficiency and bioactive-component production in *Prunella vulgaris* L. under hydroponic condition. J Plant Nutr 39:1672–1680. 10.1080/01904167.2016.1161785

